# The spatial allocation of attention in an interactive environment

**DOI:** 10.1186/s41235-019-0164-5

**Published:** 2019-04-17

**Authors:** Katherine Wood, Daniel J. Simons

**Affiliations:** Department of Psychology, University of Illinois, 603 E. Daniel Street, Champaign, IL 61820 USA

**Keywords:** Inattentional blindness, Spatial attention, Interactive environment

## Abstract

Inattentional blindness methods allow for an unobtrusive measure of the spatial distribution of attention; because subjects do not expect the critical object, they have no reason to devote attention to task-irrelevant regions in anticipation of it. We used inattentional blindness to examine the spatial allocation of attention in an interactive game in which subjects navigated through a dynamic environment and avoided hazards. Subjects were most likely to notice unexpected objects in the areas with the greatest risk of contact with a hazard, and less likely to notice equally proximal objects in inaccessible areas of the display or areas in which hazards no longer posed a threat. These results suggest that both the content of the environment and how a subject can interact with it influence the spatial allocation of attention.

## Significance

To a pedestrian, a city block is a grid of straight, walkable spaces, adjacent to a rushing river of traffic. While walking on a sidewalk, a pedestrian is not likely to take note of the speed limit for the road, or to notice empty parking spaces in front of a restaurant, unless they plan to cross the road. For a driver, navigating a city block is dictated by signs, lights, and other drivers; they are not likely to notice a canvasser with a clipboard on the sidewalk (but the pedestrian probably will), but will immediately be aware of the parking spot the pedestrian disregarded. How does the combination of an environment, our interaction with it, and the obstacles it contains influence our attention? How do the risks to our actions created by our surroundings affect our likelihood of noticing something unexpected? Our experiments used a simple road-crossing game in which subjects ferried objects from one safe sidewalk to the other, avoiding “cars” along the way. We used inattentional blindness to unobtrusively measure the spatial allocation of attention as they completed this task, and revealed a strong influence of the environment and its attendant hazards on where subjects directed their attention.

The same place can seem entirely different depending on how we move through it. When walking to our regular coffee shop, we concern ourselves with navigating around other pedestrians on the sidewalk and checking that streets are safe to cross, paying little mind to the cars passing by. Driving there places different demands on attention; we would focus on the cars, crosswalks, traffic signals, and open parking spaces, paying little heed to pedestrians on the sidewalk. When a task requires us to focus our attention on a particular region of space, we appear to ignore or filter out task-irrelevant areas.

We engage in this filtering of regions outside our focus even when performing straightforward tasks with simple displays that require no walking, or even eye movements. When subjects focused on a cross in the center of an otherwise empty display and judged which of its arms was longer, they were less likely to notice a new, unexpected object the further it appeared from the cross (Newby & Rock, 1998). Similarly, when counting how many times a subset of the moving objects in a display crossed a horizontal line bisecting the display, subjects were increasingly less likely to notice unexpected objects the further they were from the line (Most, Simons, Scholl, & Chabris, 2000; Stothart, Boot, & Simons, 2015).

Inattentional blindness methods are especially well-suited to studying the effects of proximity to the focus of attention. Because the critical object appears unexpectedly, subjects have no reason to divert attention from their primary task, or to attend to or ignore objects they might not otherwise. Other studies probing the spatial characteristics of the “attentional spotlight” do not have this advantage, instead often deliberately interfering with the primary task by cuing movement of attention away from the stimulus (e.g. Posner, Snyder, & Davidson, 1980) or employing highly confusable distractor stimuli near the focus of attention (e.g. Müller, Mollenhaur, Rösler, & Kleinschmidt, 2005). However, for the inattentional blindness tasks used to study proximity to date, the spatial layouts of the displays were arbitrary and dictated by the task. Unlike the pedestrian strolling to the coffee shop, their actions do not guide attention in these tasks. There is nothing inherent to these displays that would naturally direct attention to a particular area, and subjects are not interacting with the displays themselves beyond making judgments or counting with their eyes fixed on one spot. The role of context and task is left an open question in these particular paradigms.

Tasks in which subjects interact with an environment in some way reveal an influence of this interaction on how and where they allocate attention. When subjects are moving through a road-like setting, they show worse change detection performance while actively steering themselves compared to when they were “passengers” (Wallis & Bülthoff, 2000). However, the active “drivers”, while worse overall, detected changes better near the center of the road than changes farther from it. The demands of driving apparently narrowed the scope of attention to elements closer to the road. However, this task too relied on change detection as the primary task, which was unrelated to the act of navigating the environment.

Consistent with the effect of action demands on attention, both novice and expert drivers fixate on the road one to two seconds ahead, but the patterns of fixations vary depending on the kind of road (Underwood, Chapman, Brocklehurst, Underwood, & Crundall, 2003). On low-traffic rural roads, drivers tended to spend more time looking straight ahead. On roads with merges, they tended to check their mirrors more frequently. Although it is reasonable to assume that attention follows gaze with these drivers, and that they pay more attention to the road straight ahead when they do not have to execute any complicated maneuvers or respond to other vehicles, we cannot tell from observing patterns of eye movements alone where attention is allocated.

Studying the effects of how subjects interact with their environment on the allocation of attention requires a task with several properties: (a) unobtrusive measurement of attention; (b) sufficient freedom to make the actions seem natural; and (c) enough control to allow systematic measurement of where attention is allocated. We developed a simple road-crossing game in which subjects shuttle objects between safe zones (sidewalks), avoiding obstacles along the way and earning bonus points for speed. We use an inattentional blindness paradigm in which our primary measure is the likelihood of noticing an unexpected object as a function of its position in the display.

Across several experiments, we use this task to address a number of questions. Most importantly, how do the constraints of an environment influence the allocation of attention when all subjects need to do is interact naturally with it? Inattentional blindness tasks like ours are especially well-suited to address this question because they measure attention unobtrusively. Subjects do not have to split their attention between interacting with the display and performing an unrelated secondary task. Furthermore, because we measure attention using an unexpected object—rather than one that is always present but ignored, or a rare but not unexpected object—we can be confident that subjects are not deliberately allocating attention to the object or adopting a goal of detecting it.

Our specific implementation of an inattentional blindness task also allows us to ask whether various environmental constraints, such as the means by which subjects can travel and the behavior of hazards, influence attention and noticing. We can further examine whether the behavior of the unexpected object itself influences noticing beyond what we might predict based on the demands on attention induced by the task environment alone.

## General methods

### Subjects

The need for signed consent was waived by the University of Illinois Institutional Review Board due to the low-risk nature of the experiment. The subjects in all experiments were US-based workers recruited through Amazon’s Mechanical Turk service. We used TurkGate (Goldin & Darlow, 2013) to screen out subjects who had previously participated in experiments from our lab based on their worker ID. Subjects were directed to an external website running the experiment in Javascript, and upon finishing the experiment they received and entered a completion code to receive payment ($0.30) for the HIT (“Human Intelligence Task”, the term for the jobs posted to MTurk).

Subjects were automatically recruited in batches of up to nine using the boto3 Mechanical Turk SDK (https://github.com/boto/boto3). When we passed the recruitment threshold for an experiment, recruitment stopped and no further HITs were posted.

Results from previous studies from our laboratory using similar recruiting methods suggest that we could expect to exclude approximately 30–40% of all data collected. We set recruitment thresholds expecting to be able to use approximately 60% of the data in our final analysis for Experiments 1 and 2; however, exclusion rates were lower than anticipated, and so in Experiments 3 and 4 we recruited expecting 80% usable data. Because Experiment 5 used an unexpected object that differed substantially from the other experiments, we piloted that task prior to data collection with small groups of about 40 subjects each (both were intended to test the effectiveness of the procedures and not to estimate the effects of interest). The overall procedure in the pilot was identical to that of the main experiment. The first pilot used a slightly different version of the unexpected object. The second verified that a substantial update to the Chrome browser released just prior to launching the experiment did not cause an increase in self-reported technical issues for subjects. Based on the exclusion rates for those pilot subjects, we recruited for 70% usable data in Experiment 5.

### Materials and procedure

All experiments and analyses were preregistered on the Open Science Framework (OSF; https://osf.io/brk6t/wiki/home/). Each experiment was preregistered separately, prior to data collection for that experiment. Anonymized data, all experimental materials, analysis scripts, and preregistrations for each experiment are available on OSF.

**Fig. 1:**
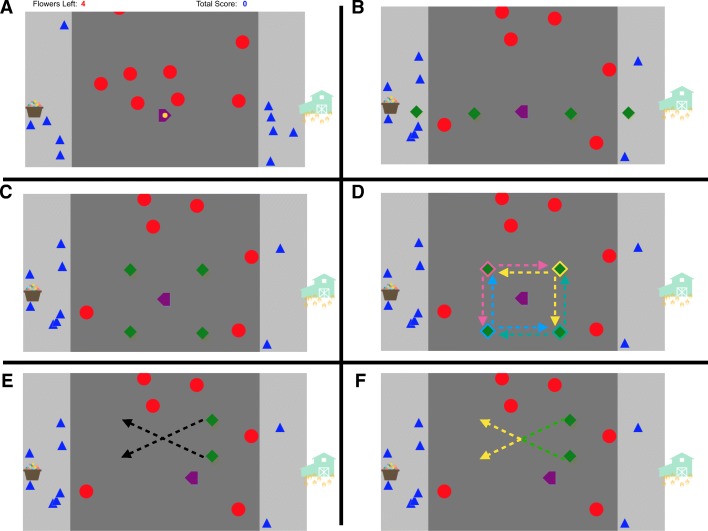
Experimental setup. **a** A screenshot of the game, after a seed has been picked up. **b** The four possible unexpected object positions in Experiment 1. **c** The four possible unexpected object positions in Experiment 2. **d** The four possible starting positions of the unexpected objects in Experiment 3. The *dotted lines* show the possible trajectories and are color-coded to show which unexpected objects can take which trajectories. **e** The two starting positions and corresponding trajectories for the unexpected objects in Experiment 4. **f** The two starting positions and corresponding trajectories for the unexpected objects in Experiment 5. The color of the *dotted lines* corresponds to the color of the unexpected object at that point in its motion (the unexpected object could also start yellow and turn green)

Prior to the experiment, subjects were shown an information screen that provided experimenter and institutional review board contact information. It explained that their responses would be anonymous, described how their data would be used, and noted that their participation was voluntary. They were then presented with an instruction screen explaining how to play the game, and after they clicked through it the game loaded and began to run. The play area consisted of a road, bordered on either side by sidewalks (for a screenshot of the game, see Fig. 1a; for detailed parameters of the game objects, see Table 1—note that because subjects completed the experiment on their own devices, screen size and viewing distance could not be controlled, so all distances and object sizes are given in pixels^1^). Blue triangle “pedestrians” appeared once every 400 ms, starting off-screen either above or below the display at a random horizontal position within the bounds of the sidewalk, and traveled either top-to-bottom or bottom-to-top either quickly or slowly. Up to ten pedestrians across both sidewalks were on screen at once. Red circle “cars” emerged from the top of the screen, traveling top-to-bottom at a randomly selected speed. Cars appeared continuously throughout the task and up to ten could be on screen at once. On the right side of the play area was a barn, and on the left, a basket of seeds.

**Table 1:** Appearance and behavior details for the objects used in the game; these parameters were consistent across experiments

Object	Maximum horizontal × vertical size (in pixels)	Color(s)	Speed(s) (in pixels/second)
Roadway	600 × 500	Dark gray (#777777)	NA
Sidewalks	150 × 500	Medium gray (#C1C1C1)	NA
Seed basket and barn	220 × 200	Cartoon image	NA
Pentagon avatar	40 × 40	Purple (#800080)	180
Pedestrian triangles	30 × 30	Blue (#0000FF)	60 or 120
Car discs	44 × 44 (radius = 22)	Red (#FF0000)	60, 120, 180, or 240
Seed disc	16 × 16 (radius = 8)	#FFD700, #F08080, #FFA07A, #20B2AA, or #87CEFA	NA

Subjects controlled their avatar with the arrow keys, and it could only move in one direction. For example, when crossing from left to right, the avatar could only move to the right. While a key was depressed, the avatar moved at a constant velocity with no acceleration. The subject’s avatar started on the left side of the screen at a fixed vertical distance of 300 pixels from the top of the game area, pointing toward the seed basket; they could only move right-to-left until they touched the seed basket, at which point their avatar picked up a randomly colored seed and reversed to point towards the right side of the play area. Subjects could then only move left-to-right until they reached the barn. Subjects “planted a flower” when they carried a seed across the road and touched the barn on the opposite side, earning points equal to 50,000 divided by the number of milliseconds they took to cross, or 1, whichever number was larger. Subjects had to plant five flowers in total to complete the task. If they contacted a car, their position was reset to the middle of the sidewalk from which they had begun that crossing. During the crossing either two or three crossings prior to the final one, an unexpected object appeared. The precise behavior of the unexpected object varied by experiment, but it was always a green (#008000) 40 by 40 pixel diamond and appeared abruptly in the display (i.e., a sudden onset). The primary question asked in all experiments was whether or not participants noticed this unexpected object as a function of its position and behavior.

After they finished the game, subjects were asked whether or not they noticed anything new that was not a game object. Regardless of their professed noticing, they then were asked: (1) whether the new object was moving, (2) in which direction the object was moving, (3) what color the object was (red, green, blue, purple, yellow, gray, black, white, or brown), and (4) what shape the object was (rectangle, triangle, diamond, circle, cross, T-shaped, L-shaped, B-shaped, or V-shaped). For experiments in which the unexpected object did not move, subjects were also asked about its location, either relative to the screen (right or left side) or relative to the subject’s avatar (above or below), depending on the experiment. Finally, subjects were asked to select their age range, gender, whether their vision needs correction and if they were wearing it during the experiment, the status of their color vision, the number contained in Ishihara plate 9 (Ishihara, 1990), whether they had experienced any technical difficulties during the game, and whether they had prior experience with a similar inattentional blindness task. After submitting their final response, subjects were presented with a completion code and told to return to Mechanical Turk to enter the code and receive payment.

### Analysis software

All analyses were conducted in R version 3.5.1 (R Core Team, 2018) using packages ggplot2 version 3.0.0 (Wickham, 2009), stringr version 1.3.1 (Wickham, 2018), purrr version 0.2.5 (Henry & Wickham, 2017), tidyr version 0.8.1 (Wickham & Henry, 2018), and dplyr version 0.7.6 (Wickham, Francois, Henry, & Müller, 2017). Analysis scripts for each experiment were written and preregistered prior to data collection for that experiment and are available on OSF.

### Analysis procedure

For all analyses, we adopt an estimation-based approach. The target sample sizes we employed (roughly 100 per condition) allow us to estimate noticing rates within approximately ± 10% across experiments. We report point estimates for noticing rates in all conditions, along with 95% bootstrapped confidence intervals calculated via the percentile method (Efron & Tibshirani, 1993). For comparisons of interest, we also calculate difference scores and their 95% bootstrapped confidence intervals. Due to the nature of our data, we elected to use bootstrapped confidence intervals rather than standard-error intervals because bootstrapped intervals do not exceed the bounds of the data and can be asymmetric.

### Exclusion criteria

Our preregistered criteria excluded data from subjects who reported being younger than 18 years old; who reported needing vision correction but not wearing it during the experiment; who reported any type of non-normal color vision; who incorrectly reported the number in the Ishihara plate; who reported that the game lagged, froze, or had some other problem; or who reported prior experience with inattentional blindness tasks. For a detailed breakdown of the exclusions in each experiment, see Table 2.

**Table 2:** A breakdown of the number of subjects excluded by each criterion in each experiment

Experiment	Excluded for age	Excluded for vision correction	Excluded for color vision	Excluded for Ishihara plate	Excluded for technical issues	Excluded for prior IB experience	Total excluded
1	1	36	22	54	34	16	129
2	0	44	17	37	30	11	114
3	0	97	37	85	68	19	251
4	0	28	13	20	18	4	68
5	0	16	34	37	70	10	112

A subject could be excluded under multiple criteria, so the sum of the individual exclusions does not necessarily equal the total number of exclusions

## Experiment 1

If attention is guided by the demands of the environment in which it operates, it should be straightforward to predict where it will be allocated when the environment is constrained. In the game subjects play, the direction of travel is restricted to one direction—they can only move forward. Given that the risk of collision is always at or in advance of the subject’s current location, we might expect them to devote attention more to the region in front of their avatar than behind it. Similarly, because subjects must avoid colliding with objects while crossing the road, we might expect more attention directed to the regions of space nearest the subject’s avatar, in which the hazards pose the most threat, than to farther regions. As a result, we should expect more noticing for unexpected objects that appear near the subject’s avatar than far away, and more for objects appearing in front than behind the avatar. When collapsing across near and far conditions, a positive difference between noticing of unexpected objects appearing in front of the subject’s avatar versus behind would suggest that more attention is allocated to the area in the direction of travel than to the inaccessible area behind the avatar. Collapsing across in front and behind, a positive difference in noticing of nearby versus far away objects would indicate that more attention is allocated nearby the avatar than farther away, possibly in order to successfully avoid obstacles.

### Methods

A demonstration of the experiment, exactly as a subject would experience it but without any data collection, can be viewed at http://simonslab.com/game/crossing_demo.html.

#### Subjects

We aimed for usable data from 100 subjects per condition after exclusions (total target *N* = 400). We set a recruitment target of 600 subjects and collected data from 634 in total.

#### Materials and procedure

Subjects were randomly assigned to one of four conditions, each corresponding to a possible unexpected object location relative to the player: near and in front, near and behind, far and in front, or far and behind (Fig. 1b).

The unexpected object appeared either during the seventh crossing of the game, when subjects were carrying their fourth seed across the road, or on the eighth crossing, when they were returning to the seed basket to pick up the fifth and final seed (selected randomly). It was therefore random whether “in front” and “behind” corresponded to left or right. The unexpected object onset immediately when subjects crossed the midpoint of the game area (450 pixels from the edge) and remained visible for one second before disappearing. It appeared at the same vertical height as the subject’s avatar (300 pixels from the top of the game area), either 113 pixels away horizontally in the near case or 338 pixels away in the far case. In the “in front” condition, the unexpected object appeared in the player’s path, and in the “behind” condition it appeared behind the player (i.e., in the direction their avatar could not travel). In the case of the near and in front condition, subjects could overlap with the unexpected object if they moved the entire time it was onscreen. The unexpected object occluded the avatar if they happened to intersect.

### Results and discussion

Prior to analysis, we excluded data from 129 subjects (20.3% of our sample) according to the criteria in the “General methods” section; 130 subjects remained in the “far behind” condition after exclusions, 137 remained in the “near behind” condition, 118 remained in the “far in front” condition, and 120 remained in the “near in front” condition. For our primary analysis, we coded subjects as having noticed the unexpected object if they correctly reported noticing something other than a game object, reported that it was not moving in response to both questions about the object’s motion, and correctly reported which side of the screen (right or left) the object appeared on.

The noticing rates for the unexpected objects conform to the expected allocation of attention based on the demands of the display and the task. Subjects rarely noticed the “far behind” unexpected object, at 8.5% (95% CI [4.6, 13.1]), but noticed the “near behind” object 47.5% (95% CI [39.4, 56.2]) of the time. Noticing rates were higher for the unexpected objects that appeared in front of the subjects’ avatar, with the “far in front” object noticed 38.1% (95% CI [29.7, 46.6]) of the time and the “near in front” object noticed 69.2% (95% CI [60.8, 77.5]) of the time.

**Fig. 2:**
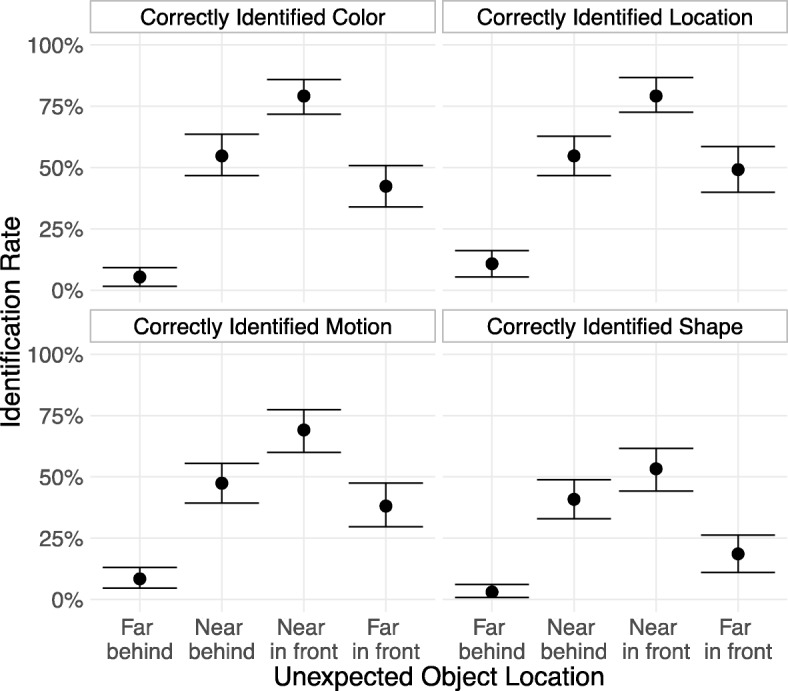
Rate at which subjects who reported seeing a new object successfully identified the unexpected object’s features, broken down by each possible object position. Error bars are 95% bootstrapped confidence intervals. To be counted as correctly identifying a feature of the unexpected object, subjects first had to report noticing something new, and: for color, report that the new object was green; for location, report which side of the screen the object was on; for motion, report that the object was not moving; and for shape, report that it was a diamond

An exploratory follow-up analysis examined whether the pattern of results differed if we counted a response as correct only following accurate identification of each of the unexpected object’s features. We found no difference in the pattern of results regardless of the feature we required to be correctly identified (Fig. 2).

People were more likely to notice unexpected objects that appeared near to their avatar than objects that appeared far away (a difference of 35.0 (95% CI [27.1, 43.0]) percentage points, collapsing across in front and behind). There was a similar, 25.3 (95% CI [16.9, 33.0]) percentage point advantage for objects that appear in front of the subject’s avatar versus behind it. It seems that people allocate their attention in response to the constraints of the environment, with most of the attention directed near their avatar and in the direction of travel.

## Experiment 2

The results of Experiment 1 confirmed that attention was allocated in response to environmental constraints. Subjects could only move in one direction, and unexpected objects were more likely to be noticed when they appeared in the path of the avatar’s motion than when they appeared behind the avatar. Similarly, unexpected objects were more likely to be noticed when they appeared near the avatar than when far from it.

One question we might ask is whether this near-versus-far advantage results from the threat of collisions. Although unexpected objects whose features match those of threatening objects are not noticed more often than objects with features associated with neutral or rewarding objects in a game context (Stothart, Wright, Simons, & Boot, 2017), the hazards in our task might influence the spatial allocation of attention given their immediate consequences for action. If so, there should be differences in noticing rates for equidistant unexpected objects depending on where they appear relative to the subject’s avatar. An unexpected object that appears in front of and above the avatar, where there is the greatest danger of a collision (because the cars move from the top of the display to the bottom), ought to be noticed more often than an object the same distance away but beneath the player, where the risk of a collision has passed.

Experiment 2 uses the same methods as Experiment 1 to explore whether there is an above/below difference in noticing, similar to the near/far and in-front/behind differences observed in Experiment 1. When we collapse across the above/below conditions and examine the difference in noticing for the in front versus behind unexpected objects, we expect the same positive difference we observed in Experiment 1. Additionally, if more attention is directed to the high-risk areas above the avatar than to the areas below it, we expect a positive difference in noticing for unexpected objects appearing above versus below (collapsing across in front and behind conditions).

### Methods

A demonstration of the task may be viewed at http://simonslab.com/game/updown_demo.html.

#### Subjects

We aimed for usable data from 100 subjects per condition, for a total of 400 subjects after exclusions. We recruited 540 subjects in total.

#### Materials and procedure

Experiment 2 used the display and task described in the “General methods” section and all details are identical to Experiment 1 except for the position of the unexpected object. In Experiment 2, the unexpected object could onset 122 pixels in front of or behind the player, and 122 pixels either above or below the player (Fig. 1c) for a total of four conditions. If the avatar moved the entire time the unexpected object was onscreen, it would come level with an unexpected object that appeared in front of the avatar, but the avatar would not pass it before it offset. The post-game survey asked whether the unexpected object appeared above or below the subject’s avatar (rather than what side of the screen it had appeared on as in Experiment 1).

### Results and discussion

**Fig. 3:**
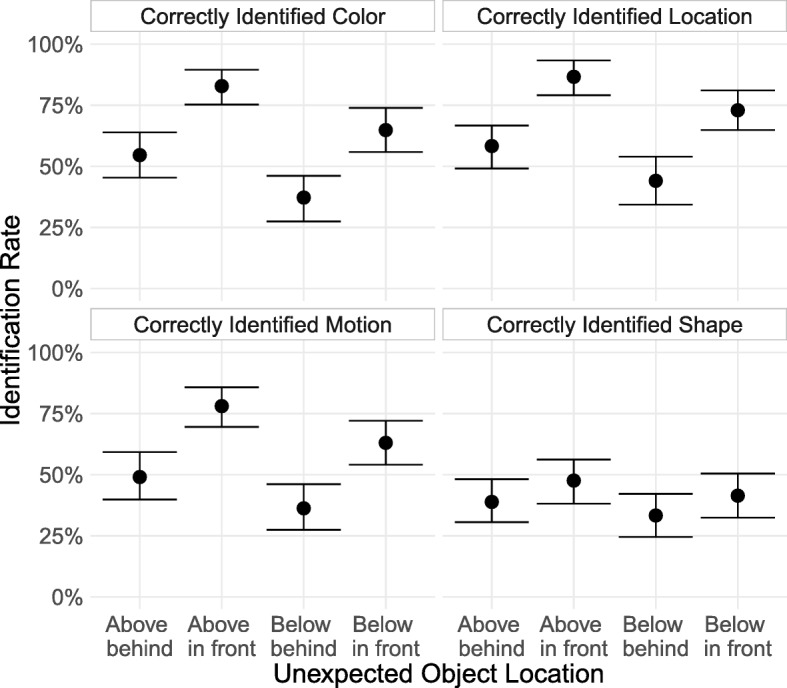
Rate at which subjects who reported seeing a new object correctly identified the unexpected object’s features, broken down by each possible position. Error bars are 95% bootstrapped confidence intervals. To be counted as correctly identifying a feature of the unexpected object, subjects had to report noticing something new, and: for color, report that the new object was green; for location, report whether the object appeared above or below their avatar; for motion, report that the object was not moving; and for shape, report that it was a diamond

We excluded data from 114 subjects (21% of our sample) prior to analysis. After exclusions there were 108 subjects in the “behind above” condition, 102 in the “behind below” condition, 105 in the “in front above” condition, and 111 in the “in front below” condition. As with Experiment 1, we classified subjects as having noticed the unexpected object if they reported noticing something new, said it was not moving, and correctly reported whether it had appeared above or below them. Among subjects reporting something new, the pattern of results was similar regardless of which feature we required to be correctly identified (Fig. 3).

Overall, we found the same in front versus behind advantage as in Experiment 1, with unexpected objects appearing in the path of travel noticed 27.5 (95% CI [18.6, 36.1]) percentage points more than objects appearing behind the avatar (collapsing across the above and below conditions). We also found a 13.2 percentage point advantage (95% CI [3.8, 22.5]) for objects above versus objects below.

Noticing rates varied with unexpected object location. When the object appeared behind the subject’s avatar, it was noticed more when above the avatar (49.1%; 95% CI [38.9, 59.3]) than below it (36%; 95% CI [26.5, 46.1]). When the object appeared in front of the avatar, it was noticed more when above (78.1%; 95% CI [70.5, 85.7]) than when below (63.1%; 95% CI [55.0, 71.2]) the avatar.

The increased noticing for objects that appear above and in front of the subject’s avatar suggests that subjects are allocating their attention more heavily to areas in which they are at risk of colliding with a harmful object. Indeed, the “above and in front” unexpected objects had the highest noticing rate of any unexpected object in Experiment 1 or 2, even more so than objects that appeared directly in the path of travel. Subjects seem to be sensitive to the demands of the environment necessary for completing their task and they direct their attention accordingly.

## Experiment 3

In Experiments 1 and 2, when participants performed a dynamic, goal-directed task in which they navigated an avatar through an obstacle-filled display, they monitored the space in front of their avatar more than the space behind it, the space above more than the space below, and nearby locations more than far away ones. Where an unexpected object appears relative to a subject’s avatar has a substantial impact on its likelihood of being noticed.

The unexpected objects in Experiments 1 and 2 were all static and occupied the same region of space the entire time they were on screen. These static objects allow for a measure of the “attention spotlight” (Posner et al., 1980), but they do not allow an assessment of the dynamics of attention over time. In particular, the unexpected objects remain stationary while the avatar—and, presumably, the focus of attention—moves, changing the position of the objects relative to attentionally relevant areas over time. Static objects do not provide a clear understanding of how objects moving in and out of the attended region interact with attention. Does the distribution of attention act only on space, so that if an object travels into a region of greater attentional relevance, it will be noticed more often, regardless of where it originated? Or does the distribution of attention apply not just to the space, but to all of the objects contained within it? That is, will an object that originates in an attentionally irrelevant area be noticed less often, even when it travels into an area of greater attention?

Results from early selective looking studies suggest that an object is no more likely to be noticed by virtue of passing into an attended area. In a task requiring subjects to count basketball passes between dark-shirted players and ignore white-shirted ones, subjects failed to notice a woman with an umbrella walking through the video, even when playback was stopped at a moment when the woman appeared to be kicking the tracked basketball (Becklen & Cervone, 1983). Passes frequently went through the woman and she often overlapped with monitored players, but noticing rates never exceeded 35%. However, as with other dynamic inattentional blindness tasks, subjects in this task passively observed the display, and the requirement to monitor three players across the screen precluded the narrow spatial distribution of attention we observed in Experiments 1 and 2 using our game task. The motion of the unexpected object may have a greater impact on noticing in our framework.

Experiment 3 presented moving unexpected objects in the same road-crossing task to examine these questions. Experiments 1 and 2 revealed substantial differences in the likelihood of subjects detecting unexpected objects depending on where they appeared; Experiment 3 explored whether similar differences exist for objects that onset in relevant areas and offset in irrelevant ones (or vice versa). Collapsing across the unexpected object’s trajectory allows us to verify whether the overall advantage for unexpected objects appearing above versus below and in front versus behind still emerge. Collapsing across position, we can determine the difference in noticing rates for unexpected objects that start in an irrelevant area and move into a relevant one (or the reverse) for horizontally and vertically moving objects. A positive difference would suggest an advantage for objects that move into a relevant area, a negative difference would suggest an advantage for objects that start in a relevant area, and no difference would suggest that the type of motion does not have a substantial impact on noticing.

### Method

A demonstration version of the task with no data collection may be viewed at simonslab.com/game/transit_demo.html.

#### Subjects

We recruited 1000 subjects to get 100 per condition for eight conditions. Subjects were recruited according to the procedure outlined in the “General methods” section, and we collected 1082 in total.

#### Materials and procedure

The gameplay aspect of the task was unchanged from the general method; the only adjustment to the method concerned the unexpected object. The unexpected objects appeared in one of the four positions used in Experiment 2; 122 pixels above or below the center of the display, and 122 pixels above or behind the center of the display. However, rather than appearing when the player had crossed the halfway point of the display, they appeared when the player had traveled 360 pixels (90 pixels shy of the halfway point). The unexpected object appeared and began moving at 240 pixels per second, traveled 244 pixels in a particular direction, and was onscreen for 1016 milliseconds. Because the unexpected object moved slightly faster than the avatar and appeared when the avatar had not yet reached the midpoint of the screen, a horizontally moving unexpected object would spend half of its time in front of the subject’s avatar and half behind (assuming the avatar moved continuously while the unexpected object was onscreen), and a vertically moving unexpected object would spend half its time above the avatar and half below. Due to the positions and speeds of the objects, the unexpected object would always offset at least 60 pixels ahead of the avatar in the horizontal direction regardless of how much the avatar moved while the object was on screen.

The unexpected object could travel either horizontally (e.g., top-right to top-left) or vertically (e.g., top-right to bottom-right) from its starting position. Two directions of travel crossed with four starting positions yielded eight conditions in total (Fig. 1d). As before, the probe appeared either when the player was crossing left-to-right (the seventh crossing) or right-to-left (the eighth crossing).

In the post-game survey, subjects were asked about the motion of the unexpected object and its appearance, but were not asked where on screen the object appeared.

### Results and discussion

We excluded data from 251 subjects (23% of our sample) from our analysis using the same criteria as prior experiments. See Table 3 for the number of subjects included in each condition.

**Table 3:** Number of subjects in each condition in Experiment 3 following exclusions

	Behind, above	Behind, below	In front, above	In front, below
Horizontal motion	118	98	104	97
Vertical motion	106	98	105	105

In this experiment, to be counted as having noticed the unexpected object for the primary analysis, subjects had to (a) report having noticed a new object, (b) report that it was moving, and (c) correctly identify its direction of motion from a choice of five directions (up, down, left, right, or not moving).

**Fig. 4:**
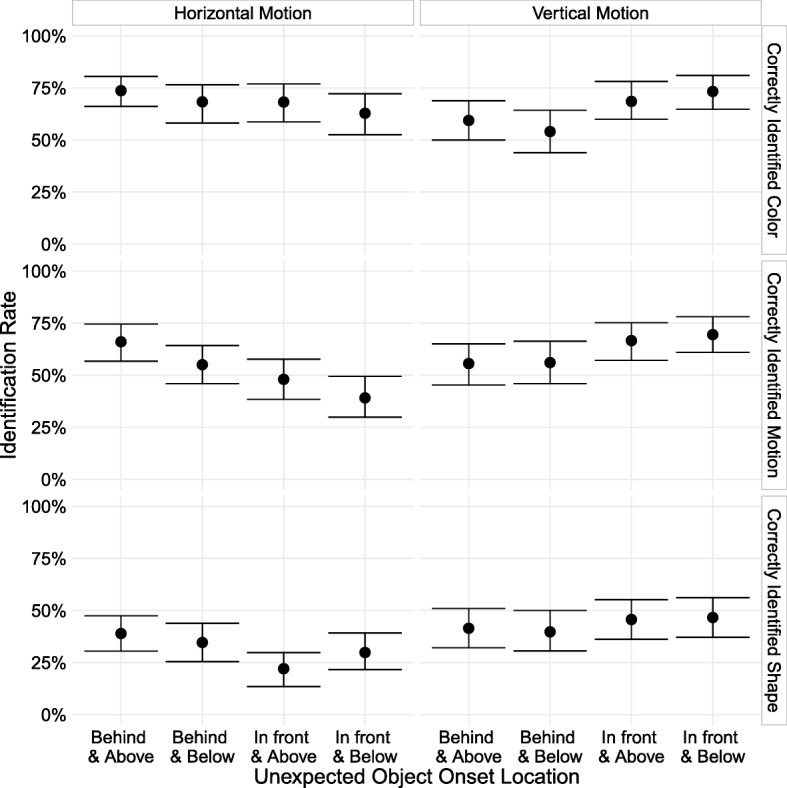
Rate at which subjects who reported seeing a new object correctly identified the unexpected object’s features, broken down by each possible position and motion trajectory. Error bars are 95% bootstrapped confidence intervals. To be counted as correctly identifying a feature of the unexpected object, subjects had to report noticing something new, and: for color, report that the new object was green; for motion, select the correct trajectory; and for shape, report that it was a diamond

Collapsing across motion direction, we observed similar location effects as Experiment 2. Unexpected objects that traveled horizontally above the subject’s avatar were noticed 10.5 percentage points (95% CI [0.8, 20.4]) more than the objects that traveled horizontally below the avatar. Objects that travelled vertically in front of the avatar had a 12.2 percentage point advantage (95% CI [2.5, 21.8]) over objects that travelled vertically behind the avatar (Fig. 4). These results replicate the patterns observed in Experiments 1 and 2 with the static object locations, once again indicating that attention is allocated according to the constraints imposed by the direction of travel and obstacle avoidance.

There was no substantial difference in noticing for objects that traveled upwards from below versus objects that traveled downwards from above when collapsing across position (an overall difference of 1.9 percentage points; 95% CI [− 7.2,11.3]). Although vertically moving objects that appeared in front of the avatar were noticed more than those that appeared behind, the upward and downward trajectories were noticed at similar rates in each case (a 2.9 percentage point difference between upwards and downwards trajectories for the in-front objects, 95% CI [− 9.5, 14.3], and a 0.5 percentage point difference for the behind objects, 95% CI [− 13.4, 13.3]).

There was a difference in noticing for objects that started behind the avatar and overtook it as they traveled horizontally compared to those that started in front and traveled towards the avatar (an overall difference of 17.3 percentage points; 95% CI [7.6, 27.2]). As for the vertical trajectories, this pattern was consistent regardless of position (an 18 percentage point difference in noticing between overtaking and passing objects moving above the avatar, 95% CI [5.4, 31.1], and a 15.9 percentage point difference for objects below the avatar, 95% CI [1.6, 29.3]).

Results for vertically moving unexpected objects did not support a difference in noticing when an object moves from an attentionally relevant area into an irrelevant one, or when it moves from an irrelevant region to a relevant one; the only major difference was the overall effect of in-front versus behind that we observed in earlier experiments.

For horizontally moving objects, the results appear consistent with greater noticing of objects that move into a relevant region from an irrelevant one, given that noticing rates were higher when the unexpected object started behind and travelled alongside the avatar. However, that pattern of motion also meant that the unexpected object spent more time near the player’s avatar if the avatar moved while the unexpected object was onscreen. While the time in front versus behind the avatar was equated, the objects that traveled towards the avatar spent much less time nearby than the one that tracked alongside it and overtook it. The large difference in noticing could be due entirely to this difference in proximity. Although motion direction is confounded with proximity within a position, we nevertheless observed the same overall above versus below advantage that we saw in previous experiments when collapsing across these motion directions.

Experiment 4 attempts to replicate the critical finding of greater noticing when an object moves from an irrelevant to a relevant region while controlling for the confound of time nearby the player’s avatar.

## Experiment 4

In Experiment 4, we used unexpected objects whose trajectories and distance to the subjects’ avatar were equated across conditions, varying only whether an object started outside of the assumedly attended region and moved into it or vice-versa. Finding a large difference in noticing of the unexpected object between the conditions (as in Experiment 3, but without the proximity confound) would indicate an effect of the unexpected object’s trajectory into or out of an attentionally relevant area on noticing.

### Methods

A demonstration of the task may be viewed at http://simonslab.com/game/xtransit_demo.html.

#### Subjects

We anticipated a 20% exclusion rate, so we recruited 291 subjects to finish with 100 per condition.

#### Materials and procedure

Methods and gameplay were identical to those described in the “General methods” section, except for a change in the motion of the unexpected object. The unexpected object appeared when the subject has traveled 360 pixels, and always appeared 122 pixels behind the player horizontally. It could start in one of two vertical locations; 122 pixels above the player, or 244 pixels above the player (Fig. 1e). The object appeared when the player was either crossing left-to-right (crossing 7 of 10) or right-to-left (crossing 8 of 10).

After onset, the unexpected object moved diagonally, traveling at four pixels per second in the x-dimension and two pixels per second in the y-dimension, traveling 244 pixels horizontally and 122 pixels vertically total. If the unexpected objects started “far” above the player’s avatar (244 pixels), it moved diagonally downward to overtake the player and finish close to them (122 pixels above and 122 pixels in front). If it started near to them (122 pixels above), it moved diagonally upward to finish farther away from them (244 pixels above and 122 in front). The two possible motion paths are reflections of each other, so distance to the player over the course of the trajectory was identical (assuming that the subjects either (a) moved at a constant rate while the probe was onscreen or (b) that players in the two conditions had similar patterns of motion while the probe was on screen). This manipulation therefore controlled for the amount of time spent nearby the player’s avatar while allowing us to test whether an unexpected object that moves into a more relevant area (the area above and in front of a player) is noticed more often than one that moves into a less relevant area (farther above the player).

Due to the unexpected object’s diagonal trajectory, when subjects were asked to report the object’s motion, they were required to select the direction they thought it moved from eight arrows (four pointing to the cardinal directions, four to the inter-cardinal directions).

### Results and discussion

We excluded data from 68 subjects from analysis (23% of our sample). As in Experiment 3, our primary criterion for noticing was correct identification of the unexpected object’s motion. Subjects had to report noticing something new, report that it was moving, and choose the correct direction of motion from an array of arrows. We had 118 subjects in the starts-far, ends-close condition and 105 in the starts-close, ends-far condition after exclusions.

**Fig. 5:**
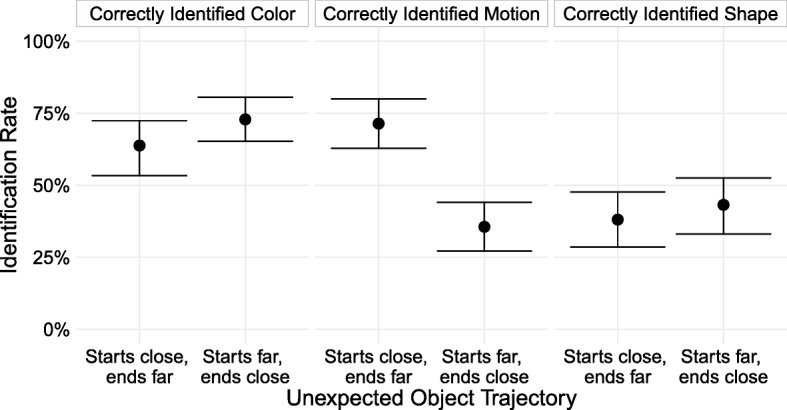
Rate at which subjects who reported seeing a new object identified the unexpected object’s features, broken down by the unexpected object’s trajectory. Error bars are 95% bootstrapped confidence intervals. To be counted as correctly identifying a feature of the unexpected object, subjects had to report noticing something new, and: for color, report that the new object was green; for motion, select the correct trajectory from the cardinal and inter-cardinal directions; and for shape, report that it was a diamond

There was a large difference in noticing between conditions. Subjects noticed an unexpected object that appeared near them and moved away 71.4% (95% CI [62.9, 80.0]) of the time, but noticed an object that appeared far from them and got closer only 35.6% (95% CI [27.1, 44.1]) of the time (a difference of 35.8 percentage points, 95% CI [23.8, 48.2]; Fig. 5). Noticing rates were similar to the approximately comparable condition in Experiment 3, in which the unexpected object started above and behind the avatar and traveled horizontally to overtake it (noticed 66% of the time).

Unexpectedly, and unlike in previous experiments, the pattern of correct identification between conditions varied across features. Although the starts-close, ends-far group was nearly twice as accurate at identifying the unexpected object’s motion as the starts-far, ends-close group, the size of the difference was not just smaller for identification of color and shape, but in the opposite direction. The starts-far, ends-close subjects correctly identified the unexpected object’s color 9.1 percentage points more than the starts-close, ends-far group, (95% CI [− 2.9, 21.2]) and correctly identified the shape 5.1 percentage points more (95% CI [− 7.5, 18.4]). Why do these groups differ in their ability to identify the motion direction, but less so in their ability to identify other features of the unexpected object?

One possibility is that the time course of noticing differs between the two conditions. Subjects may notice the unexpected object once it draws near. If so, when it starts nearby and travels away, subjects would notice it sooner and be able to track it during the entire course of its movement. In contrast, when the object starts far away and gets closer, they may not notice it until the last moment and cannot track its path of motion over time but can identify its other features.

## Experiment 5

Experiment 5 tested whether the timing of noticing might explain the difference in motion identification between the two conditions. The study duplicated Experiment 4 with a change to allow us to determine roughly when subjects noticed the unexpected object: the unexpected object changed color halfway through its trajectory. If the difference in accuracy when reporting the unexpected object’s motion between the two conditions in Experiment 4 was due to noticing the object early versus late, we should see more subjects reporting the unexpected object’s second color in the condition in which the unexpected object appears far away and gets closer.

We expect to observe the same pattern of unexpected object feature identification as in Experiment 4: a large difference between conditions in correct identification of the motion of the unexpected object, but no such differences for shape or color identification. If the difference for motion identification results from when the unexpected object is noticed, then we should find that the subjects who reported noticing the unexpected object and could correctly identify its color are more likely to report the earlier color when the unexpected object onsets close to the avatar, and the later color when the unexpected object onsets far away from the avatar.

### Methods

A demonstration of the task may be viewed at simonslab.com/game/xcol_demo.html.

#### Subjects

We recruited 313 with the goal of 100 usable subjects per condition.

#### Materials and procedure

The gameplay was identical to that described in the “General methods” section; however, the number of required crossings was reduced from ten to eight. The median time to complete the game in Experiment 4 was roughly 4.5 min. In order to maintain a fair pay rate for the task, the gameplay portion was shortened. The unexpected object thus appeared randomly on the fifth or sixth of eight crossings; the procedure was otherwise unchanged.

The behavior and movement of the unexpected object was identical to Experiment 4. However, the unexpected object started with one of two colors, green (#1bad1b) or yellow (#cccc26). It remained that color for 24 frames, then linearly interpolated to the other color (yellow if it began as green, green if it began as yellow) over the course of ten frames, then remained its final color for 24 frames before offsetting. In the post-game survey, rather than being asked what color the unexpected object was, subjects were asked what color it was when they first noticed it. All other questions were unchanged from Experiment 4.

### Results and discussion

**Fig. 6:**
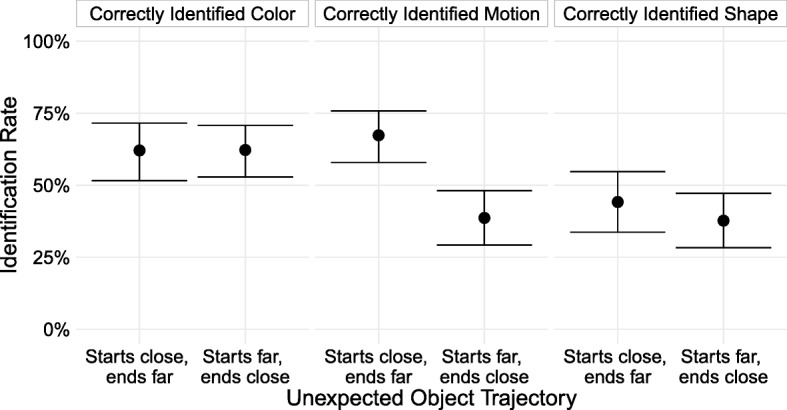
Rate at which subjects who reported seeing a new object identified the unexpected object’s features, broken down by the unexpected object’s trajectory. Error bars are 95% bootstrapped confidence intervals. To be counted as correctly identifying a feature of the unexpected object, subjects had to report noticing something new, and: for color, report that the new object was green or yellow; for motion, select the correct trajectory from the cardinal and inter-cardinal directions; and for shape, report that it was a diamond

Prior to analysis, we excluded data from 112 subjects (36% of our sample), leaving 106 subjects in the starts-far, ends-close condition and 95 in the starts-close, ends-far condition. Overall, we replicated the results of Experiment 4. Correct identification of the path of motion differed starkly between conditions, at 67.4% when it appeared nearby and moved away versus 38.7% when it appeared far away and approached (a 28.7 percentage point difference, 95% CI [15.8, 41.1]), but correct identification of the other features did not differ much between starts-close, ends-far and starts-far, ends-close (a difference of − 0.2 percentage points, 95% CI [− 13.2, 13.2], for color and 6.5 percentage points, 95% CI [− 5.9, 19.8], for shape; Fig. 6).

Among subjects who correctly reported the color of the unexpected object, there was no difference between conditions in the likelihood of reporting the first versus last color. For the start-far, end-close condition, of those who correctly identified one of the object’s colors, 33.3% reported the first color and 66.7% reported its second color. For the start-close, end-far condition, 32.2% reported the first color and 67.8% reported the second color. While the color subjects reported is not a perfect indicator of when they noticed the object—the second color may overwrite the first in memory, for instance, or some subjects who see both colors may be biased to report the last color they saw—the absence of a difference between the two conditions likely rules out large differences in the time course of noticing as the explanation for the discrepancy in motion identification.

Why, then, is one group much less accurate than the other in identifying the motion of the unexpected object if there is no difference in when they first notice it? One possibility is suggested by examining the vertical and horizontal components of the motion identification separately (Table 4). While subjects in the starts-close, ends-far condition were equally accurate on identifying the horizontal and vertical components of the unexpected object’s motion (that is, they reported that it was moving upwards just as accurately as they reported it moving right or left), subjects in the starts-far, ends-close condition were nearly twice as accurate at reporting the horizontal component compared to the vertical one. A post-hoc analysis of the motion reporting in Experiment 4 reveals an identical pattern; the reason the starts-far, ends-close group had such low accuracy is that they were much less likely to detect the vertical component of the motion. Because the unexpected object covers twice as much distance horizontally as it does vertically, the signal may be stronger for the horizontal component of the motion. The starts-far, ends-close group may merely be more uncertain, and guesses the horizontal direction (of which they might be more sure) and disregards the vertical component of the motion. This difference in groups may therefore simply reflect different response strategies under different levels of certainty, rather than any differences in attention.

**Table 4:** Identification rates for the component motion by condition for Experiments 4 and 5

Experiment	Motion type	Identified motion	Identified vertical motion	Identified horizontal motion
Experiment 4	Starts far, ends close	35.6%	39.0%	73.7%
Experiment 4	Starts close, ends far	71.4%	73.3%	73.3%
Experiment 5	Starts far, ends close	38.7%	41.5%	63.2%
Experiment 5	Starts close, ends far	67.4%	70.5%	70.5%

To be counted as noticing the motion overall, subjects had to get the motion direction correct. For the vertical component, they simply had to supply any direction that contained the correct vertical direction (e.g., ‘up-left,’ ‘up,’ or ‘up-right’ would be accepted), and for the horizontal component, any direction that contained the correct horizontal direction

## General discussion

The spatial allocation of attention conforms to the demands of the environment, even when that environment is a simple road-crossing game. When the direction of travel is restricted, and people can only travel forward, they are more likely to notice unexpected objects that appear in front of them than behind them, and are more likely to notice nearby unexpected objects than faraway ones. The hazardous objects in the game also play a role in directing attention; subjects were most likely to notice an unexpected object that appeared in front and above them—the area of the display in which the hazards posed the greatest threat. Unexpected objects were less likely to be noticed if they appeared the same distance away from the subjects’ avatar but were underneath it, corresponding to the area of the display in which the hazards could no longer collide with the subject’s avatar.

The way subjects allocated attention in this task reflected their appraisal of their ongoing actions and the display environment and not a strategy of searching for the unexpected object. Subjects were not told where to direct attention, were not informed whether they should attend to or ignore any objects in particular, and were not informed about the possibility of additional objects in the display. Even though subjects were free to approach the task however they liked, attention was concentrated to the most task-relevant areas. Not only does this show the role of environmental constraints on attentional allocation, but also demonstrates a naturalistic way to control the spatial deployment of attention without explicit direction.

While the results from the static objects reveal a clear pattern in the spatial allocation of attention in response to the environment, the data from the moving objects indicate little, if any, role of movement through these areas on noticing. Experiments 3–5 attempted to investigate the impact on noticing of objects traveling into and out of attentionally relevant areas. In Experiment 3, unexpected objects moving on vertical trajectories were noticed at the same rate, regardless of whether they onset in the attentionally relevant areas above the subject’s avatar and traveled to the less relevant area below the avatar, or vice versa. Although we observed a difference in noticing for the horizontal trajectories, these were confounded with proximity to the subject’s avatar. This difference disappeared after controlling for this confound (Experiments 4 and 5). When proximity to the avatar was equated over the unexpected object’s trajectory, subjects were equally likely to notice it whether it started in a less relevant area of the display and finished in a more relevant one or vice versa. Although there was a difference in subjects’ ability to correctly identify the object’s direction of motion between conditions, this appeared to be related to response strategies under uncertainty rather than any meaningful difference in attention. Overall, we replicated the findings for static unexpected objects with moving objects, finding more noticing for objects in front than behind, and for above than below. Across experiments, the movement behavior of the unexpected object in the display had much less of an impact on noticing than did the general region of the display in which it appeared. It does not seem to matter whether an object moves into or out of an attentionally relevant area, and something unexpected entering a closely monitored area does not attract any more attention than something leaving it.

Overall, the environment and the demands of performing the road-crossing task shaped the allocation of attention. People tend to monitor the highest-risk areas the most, and pay less attention to areas they cannot access and areas that no longer pose a threat to their actions. While unexpected objects that share features with threatening objects do not seem to be noticed more often than objects sharing features with neutral or rewarding objects in a game context (Stothart et al., 2017), threatening objects do seem to influence the spatial allocation of attention. Future studies can examine the relative contributions of object features and object locations to noticing in this sort of interactive environment. The nature of the games themselves may determine these contributions; both of these games had a strong spatial and hazard-avoidance component, which might have led participants to prioritize attending to object locations over object features.

Attention operates in a context. Most of the time, we deploy selective attention in the service of a goal. We might expect that the interaction of the structure of the environment, how we navigate through it, and what we intend to accomplish influences both how we deploy attention and what information we select versus filter from awareness. If we want to understand attention in natural tasks like driving or walking, an important first step is exploring attention in smaller-scale, easy-to-control environments. In order to draw conclusions that might generalize to more complex settings, however, we should try to avoid adding constraints that might alter how attention is deployed. For example, dual-task designs in which subjects navigate an environment while also responding to some secondary, unrelated task might mis-measure how we direct attention in the absence of such secondary goals. Inattentional blindness paradigms measure attention while subjects engage more naturally with a display or task without adding extraneous demands on attention, while still providing a naturalistic measure of what people notice.

### Constraints on generality

Space-based effects similar to those we investigate here have emerged in other inattentional blindness paradigms, run both in person (Most et al., 2000) and on Mechanical Turk (Stothart et al., 2015). We expect the overall effects we found to generalize to any task with similar constraints, and to generalize to in-person, lab-based, or online testing settings, although the particulars of the effects—what areas are emphasized, what distances are monitored, and overall noticing rates—likely will vary according to how the game environment is set up and the particulars of the navigation constraints and obstacle or hazard avoidance.

## Footnotes

1 In Javascript, a pixel refers to a CSS pixel rather than a physical pixel. CSS pixels scale automatically to the density of the display device, such that a single CSS pixel is drawn with more physical pixels on a higher-density display.
